# Biased Affective Forecasting: A Potential Mechanism That Enhances Resilience and Well-Being

**DOI:** 10.3389/fpsyg.2020.01333

**Published:** 2020-06-12

**Authors:** Desirée Colombo, Javier Fernández-Álvarez, Carlos Suso-Ribera, Pietro Cipresso, Azucena García-Palacios, Giuseppe Riva, Cristina Botella

**Affiliations:** ^1^Department of Basic Psychology, Clinic and Psychobiology, Universitat Jaume I, Castellón de la Plana, Spain; ^2^Department of Psychology, Università Cattolica del Sacro Cuore, Milan, Italy; ^3^Applied Technology for Neuro-Psychology Lab, IRCCS Istituto Auxologico Italiano, Milan, Italy; ^4^CIBER Fisiopatología Obesidad y Nutrición (CIBERobn), Instituto Salud Carlos III, Madrid, Spain

**Keywords:** affective forecasting, cognitive bias, ecological momentary assessment, psychological well-being, resilience

## Abstract

According to a growing body of studies, people’s ability to forecast future emotional experiences is generally biased. Nonetheless, the existing literature has mainly explored affective forecasting in relation to specific events, whereas little is still known about the ability to make general estimations of future emotional states. Based on existing evidence suggesting future-oriented disposition as a key factor for mental health, the aims of the current study were (1) to investigate the relationship between negative (NA) and positive (PA) affective forecasting biases and perceived psychological well-being, and (2) to explore whether positively biased predictions are associated with resilience and foster one’s skills to cope with stressful events. To do so, we asked 85 undergraduate students to forecast PA and NA over 2 weeks, as well as to report their daily affect through a web-based Ecological Momentary Assessment. According to the results, positively biased PA forecasting (i.e., overestimating positive emotional states) was associated with greater perceived psychological well-being and higher resilience. When high levels of stress were experienced, participants holding an optimistic, yet biased, estimation of future PA were more likely to successfully manage stressors, thus maintaining lower levels of NA and higher levels of positive emotions. We suggest that positively biased PA forecasting is an adaptive cognitive distortion that boosts people’s resilience and mental health, thus opening new avenues for the promotion of psychological well-being.

## Introduction

As terms draws to a close and summer vacations stretch out ahead, people start to mentally imagine the upcoming holidays. For instance, they visualize themselves sleeping until late, having a brunch with some friends or leaving for a tropical destination. Beyond envisioning activities, people spontaneously imagine their own future emotions (Staats and Skowronski, 1992). That is, how happy and relaxed they will feel while taking a break from work, or the excitement they will experience while visiting a new place. As evidenced by a long tradition of research, people are indeed used to mentally time travel, and they always try to imagine and predict future emotional experiences (Kahneman and Snell, 1990; Gilbert et al., 2002; Gilbert and Wilson, 2009).

Despite some sort of insight is likely to exist (Buehler and McFarland, 2001; Wirtz et al., 2003), research generally suggests that inaccuracy between forecasted emotional states and future experiences is frequent: People are not good at forecasting feelings, and the affective states they anticipate do not match the actual future experience (Wilson and Gilbert, 2003). Sources of errors in affective forecasting may be connected either to the time at which the prediction is made or to the actual experience (Wilson and Gilbert, 2003). Regardless of the type of error, the result is a bias in affective forecasts. In this sense, the literature has shown that, while people are usually quite accurate at forecasting the valence of future emotional experiences (i.e., negative or positive) or the specific emotions they will experience (e.g., anger or fear) (Wilson and Gilbert, 2003), they are quite biased at estimating emotional intensity and duration, thus leading to the so-called durability bias (i.e., the tendency to overestimate the duration of an emotional reaction) (Gilbert et al., 1998) and impact bias (i.e., the tendency to overestimate the impact of a future event) (Gilbert et al., 2002; Wilson et al., 2003).

To date, a body of studies supports the idea that affective forecasting represents an important cognitive process, and predicting future feelings is an essential source of information to drive behaviors (Mellers et al., 1997; Crawford et al., 2002; DeWall et al., 2014). Accordingly, people use affective information to make judgments and take decisions about the future (Schwarz and Clore, 1983; Taquet et al., 2016; Colombo et al., 2020). In addition, there is also evidence supporting that affective forecasting is a regulatory process, that might serve as a resilience source in the presence of difficulties. Specifically, anticipating future feelings would be a future-oriented strategy to regulate emotions (Goodhart, 1985), which would lead people to directly or indirectly behave in order to match or change the forecasted emotional experience (Persson and Sjöberg, 1985). In that direction, Totterdell et al. (1997) asked thirty participants to predict daily and weekly mood, as well as to annotate daily affect at the end of the day. Results showed that, regardless of the presence of daily hassles, mood was more likely to improve when participants expected it to improve (i.e., when they predicted that they would have experienced a better mood), thus supporting the hypothesis of affective forecasting as a regulatory process and suggesting that mood forecasts may be considered “[…] *as part of a process that exerts some mental control over mood*” (Totterdell et al., 1997).

Based on the previous literature, it seems plausible that the way people anticipate affective states can have repercussions on different aspects of life, such as happiness and well-being (Dunn et al., 2007a; Gilbert and Wilson, 2009; Buchanan et al., 2019; Nasso et al., 2019), physical and mental health (Sieff et al., 1999; Riis et al., 2005), and interpersonal relations (Dunn and Laham, 2006). Consequently, biases in affective forecasting, either positive or negative, may entail several consequences for mental health. Indeed, positive illusions such as favorable self-evaluations, exaggerated perception of control, and unrealistic optimism have been shown to boost happiness and well-being (Taylor and Brown, 1988, 1994; Brookings and Serratelli, 2007). These cognitive biases are likely to increase the perception of owning successful copying skills (Brown, 1993), which in turn enhances motivation and enthusiasm while carrying out actions (Taylor and Gollwitzer, 1995). Similarly, a positive future-oriented disposition and openness to the future (i.e., having positive expectations and a general disposition of acceptance toward the future) have been shown to be protective factors for mental health and to be positively associated with well-being (Weinstein, 1980; Mikus et al., 2017; Botella et al., 2018).

In the present study, we aimed to explore affective forecasting in a sample of undergraduate students. Contrary to the previous literature that mainly focused on predicting emotions in relation to a specific future event, we explored affective forecasting as a future-oriented disposition in healthy individuals by asking for general future affective estimations. The main objective was to disentangle the association of affective forecasting with well-being and resilience. To do so, we asked 85 participants to forecast positive (PA) and negative (NA) affect over 2 weeks, and we monitored experienced daily mood by means of a web-based Ecological Momentary Assessment (EMA) design, which has been shown to be an adequate methodology to capture emotional dynamics in daily life (Colombo et al., 2019a, b).

First, we hypothesized that people with a more optimistic view of future affect and who tend to overestimate PA will show greater well-being. No significant association is expected in relation to NA forecasts, because overestimating negatively valenced emotions is known to be either an evolutionary rather than maladaptive copying mechanism (Miron-Shatz et al., 2009), or the consequence of a negative bias associated with anxiety and depressive conditions (Mathersul and Ruscio, 2019), which were excluded from the current study. Second, and in line with the previous hypothesis, we expected that PA but not NA forecasts will be associated with resilience. More specifically, we hypothesized that PA under-estimators would be less resilient than PA over-estimators. Finally, we hypothesized that biased PA forecasts would moderate the impact of stress on affect, consistent with the idea that holding positive expectations about the future represents a further source of resilience to cope with daily events.

## Methods

We reported how we determined our sample size, all data exclusions (if any), all manipulations, and all measures in the study (Simmons et al., 2012).

### Sample

The sample size was calculated considering the correlations as the main analyses of the study. Assuming an overall moderate effect size of 0.3 (correlation), a significance level of 5%, a statistical power of 80%, and a bilateral contrast, the sample size calculation resulted in a sample of *n* = 82. Calculations were made with G^∗^Power (Faul et al., 2007).

In total, 91 undergraduate students were recruited via online advertisements at the Jaume I University (Castellon, Spain). Participants with a score above 14 on the Patient Health Questionnaire (PHQ-9) (Kroenke et al., 2001) and/or the Generalized Anxiety Disorder (GAD-7) (Spitzer et al., 2006) were excluded from the study (i.e., individuals with moderate/severe clinical conditions). Accordingly, there is evidencing showing that patients suffering from Major Depressive Disorder (MDD) or Generalized Anxiety Disorder (GAD) are negatively biased in affective forecasting (Wenze et al., 2012; Mathersul and Ruscio, 2019), which would make their inclusion together with non-clinical individuals problematic. Accordingly, 6 participants were excluded, thus leading to a final sample of *n* = 85. The sample was composed of 72 females and 13 males, and their mean age was 20.81 years (SD = 2.26). In our sample, the PHQ-9’s internal consistency was α = 0.73, whereas the GAD-7’s internal consistency was α = 0.82.

This study was approved by the ethics committee of the Jaume I University (Spain) (certificate number: CD/57/2019; reference: 41EA95C7D3C8747F0A37), and informed consent was obtained from all participants.

### Measures

#### Forecasted Positive and Negative Affect

Participants were administrated the Spanish adaptation (Díaz-García et al., 2020) of the Positive and Negative Affect Schedule (PANAS) (Watson et al., 1988). The PANAS is composed of 10 items to measure PA and 10 items to assess NA. Previous research has shown the validity and reliability of the questionnaire (Sandín et al., 1999). In the present study, the original instructions “*Indicate the extent you have felt this way over the past week*” were changed to “*Indicate the extent you think you will feel over the next two weeks*” to evaluate forecasted as opposed to retrospective affect. In our sample, both the PA and the NA subscales showed good internal consistency (PA: α = 0.91; NA: α = 0.78).

#### Psychological Well-Being

Psychological well-being was assessed using the Spanish adaptation (Díaz-García et al., 2020) of the Ryff’s Psychological Well-Being Scale (Ryff and Keyes, 1995; Ryff, 2005), which explores six different dimensions of psychological well-being: Autonomy (i.e., independence from external judgments and social prejudices: “I have confidence in my opinions, even if they are contrary to the general consensus”; “I judge myself by what I think is important, not by the values of what others think is important”), environmental mastery (i.e., the ability to take advantage of the environment to achieve personal goals: “I am quite good at managing the many responsibilities of my daily life”; “In general, I feel I am in charge of the situation in which I live”), personal growth (i.e., the sense of continuous self-improvements thanks to life experiences: “For me, life has been a continuous process of learning, changing, and growth”; “I have the sense that I have developed a lot as a person over time”), purpose in life (i.e., the sense of meaning in life, owning clear personal values and life goals: “I have a sense of direction and purpose in life”; “I enjoy making plans for the future and working to make them a reality”), positive relations (i.e., satisfactory and trusting relationships, as well as empathetic and warm attitude toward others: “People would describe me as a giving person, willing to share my time with others”; “I know that I can trust my friends, and they know they can trust me”), and self-acceptance (i.e., positive attitude toward the current and past self, as well as acceptance of both positive and negative personal qualities: “When I look at the story of my life, I am pleased with how things have turned out”; “When I compare myself to friends and acquaintances, it makes me feel good about who I am”). This scale has shown good psychometric properties (van Dierendonck, 2004). In our sample, all subscales demonstrated good internal consistency, except for autonomy and environmental mastery (self-acceptance: α = 0.87; positive relation: α = 0.83; autonomy: α = 0.64; environmental mastery: α = 0.67; personal growth: α = 0.83; purpose in life: α = 0.78).

#### Resilience

Resilience was assessed using the Spanish adaptation (Notario-Pacheco et al., 2011) of the 10-item Connor-Davidson Resilience Scale (CD-RISC10) (Campbell-Sills and Stein, 2007), a self-report scale with good psychometric properties (Singh and Yu, 2017; Shin et al., 2018) that measures resilience over the previous 30 days (“I can deal with whatever comes my way”; “I think of myself as a strong person when dealing with life’s challenges and difficulties”). In our sample, the CD-RISC10 showed high internal consistency (α = 0.85).

#### Openness to Future

The Openness to the Future Scale (OFS) is a 10-item self-report questionnaire that measures orientation toward the future, including positive expectations, a sense of competence to cope with daily events, and the acceptance of what can’t be predicted. Some examples include: “I calmly accept that good and bad things will happen to me in life”; “I am very excited about future opportunities and challenges”; “I feel hopeful about what the future may bring.” This scale has shown good psychometric properties both in community and clinical samples (Botella et al., 2018). In our sample, the OFS showed good internal consistency (α = 0.80).

### Ecological Momentary Affect (EMA) Measures

At each daily evaluation, participants were asked to complete three 100-point numerical scales (0 = not at all; 100 = extremely) evaluating momentary PA (“*To what extent are you experiencing positive emotions at this moment?*”), momentary NA (“*To what extent are you experiencing negative emotions at this moment*?”), and momentary stress (“*How would you rate your current level of stress?*”). Participants were also asked to rate the momentary level of seven positive emotions (happiness, fun, hope, serenity, excitement, pride, gratitude) using a 1-5 Likert scale (“*To what extent are you experiencing the following positive emotions at this moment?*; 1 = not at all; 5 = extremely). The sum of the seven scales reflected the momentary level of positive emotions.

### Procedure

Participants were recruited via poster advertisements at the Jaume I University (Castellon, Spain). Students interested in the study were invited to the laboratory in order to receive more information about the investigation. Participants who met the inclusion criteria were invited to sign the informed consent and to complete the affective forecasting measure with the PANAS.

Repeated daily assessments were collected by means of Qualtrics, a web-based platform that allows to create and send customized online surveys at specific time points during the day. In the present study, participants were semi-randomly prompted three times a day for 2 weeks (between 9:30 – 14:00; 14:00 – 18:30; and 18:30 – 23:00) by means of an email. After receiving the notification, participants had 60 min to enter the weblink and complete the evaluation.

At the end of the study, participants returned to the laboratory and completed the following questionnaires: The Ryff’s Psychological Well-being Scale, the CD-RISC and the OFS. Additionally, participants were asked whether something significant unexpectedly happened in the previous 2 weeks. This included any sudden and unforeseen positive and/or negative event that significantly affected their mood, thoughts, or behaviors. This question was introduced in order to exclude participants that, during the study, experienced an event that was impossible to anticipate (such as a sentimental breakup, the death of a closer person, or being hired at a new job), thus creating a biased mismatch between the predicted and experienced affect. However, no participant reported such significant events and there was no need for exclusion. A remuneration of 10 euros was given to participants who completed more than 60% of the EMA assessments.

### Data Analysis

A summary of all the variables included in the analysis and their abbreviations is reported in Table 1. Forecasted affect refers to the PANAS-PA and PANAS-NA subscale scores collected at baseline. Experienced affect refers to mean PA and NA levels experienced during the 2-week EMA, and it was obtained by calculating the mean of the 42 possible PA and NA assessments for each participant. Besides, EMA scores refer to the 42 possible NA, PA, positive emotions and stress repeated assessments collected throughout the 2-week study.

**TABLE 1:** Summary of all the variables included in the analysis and their abbreviations.

**Abbreviation**	**Variable**
Forecasted PA	Anticipated PA – PANAS at baseline
Forecasted NA	Anticipated NA – PANAS at baseline
Experienced PA	Global average of EMA-PA assessments
Experienced NA	Global average of EMA-NA assessments
EMA-PA	PA repeated EMA assessments
EMA-NA	NA repeated EMA assessments
EMA-Stress	Stress repeated EMA assessments
EMA-positive emotions	Positive emotions repeated EMA assessments
Delta PA	(Forecasted PA – Experienced PA)
Delta NA	(Forecasted NA – Experienced NA)

To distinguish between future affect overestimation or underestimation, delta scores were computed. To have the same range of scores for forecasted (PANAS: 1-to-5 Likert scale) and experienced affect measures (EMA: 0–100 scale), PANAS values were transformed to Percent of Maximum Possible (POMP) Scores (Cohen et al., 1999; Fischer and Milfont, 2010). POMP scores express raw scores in terms of the maximum possible score and can range between 0 and 100, thus facilitating the comparison of data when scales and scoring methods are not consistent. POMP scores are calculated as follows: 100 × (raw-min) / (max-min), with min and max indicating the lowest and highest scores possible according to the scale adopted. POMP scores of forecasted affect were calculated as follows: POMP scores: 100 × (raw - 10)/(50 - 10). Delta scores were therefore computed as follows: Delta = (POMP forecasted affect – experienced affect). Positive scores reflected future affect overestimation, whereas negative scores reflected future affect underestimation.

Correlation analyses were conducted to explore the association between forecasted and experienced NA, and between forecasted and experienced PA. Moreover, Generalized Estimating Equations (GEEs) with an unstructured correlation matrix structure and Huber–White standard error estimates were used, introducing forecasted PA and NA as predictors of daily EMA-NA and EMA-PA scores. GEEs are designed to examine longitudinal repeated-measures data. Furthermore, GEEs are adequate to draw inferences by considering not only variations in affective experience over time within individuals, but also variations in affective experience between individuals (Liang and Zeger, 1986; Pavani et al., 2016). Forecasted and experienced PA (Paired sample *t*-test) and NA scores (Wilcoxon Signed Ranks Test) were compared to test the participants’ ability to predict future affect. Also, delta scores distribution was explored, and their association with depressive and anxiety symptoms was investigated.

To confirm the first hypothesis, correlation analyses were conducted to explore the association between forecasted/experienced affect, delta scores, well-being, and openness to the future. GEEs with an unstructured correlation matrix structure were used introducing forecasted NA, forecasted PA, daily EMA-PA and daily EMA-NA simultaneously as predictors of psychological well-being.

To explore the association between affective forecasting and resilience, correlation analyses were conducted. Besides, multiple linear regressions were performed using well-being measures as dependent variables and resilience as the independent variable; in a second block, delta scores were included to explore significant improvements in the model.

Consistent with the third hypothesis, we performed GEEs with an unstructured correlation matrix structure and Huber–White standard error estimates including delta scores, daily EMA-stress scores and the interaction term as predictors of daily affect.

## Results

### Forecasted and Experienced Affect

An overview of the recruited sample is reported in Table 2. Overall, high compliance was obtained (*M* = 80.47%; SD = 18.44%), considering previous research exploring the extent to which participants tend to answer EMAs (Colombo et al., 2018; Van Genugten et al., 2020). Compliance was associated with depressive (*r* = −0.21, *p* = 0.05) and anxiety symptoms (*r* = −0.21, *p* < 0.05), but not with age (*r* = 0.18, *p* = 0.11).

**TABLE 2:** Detailed information about the recruited sample and affect measures (GAD-7: Generalized Anxiety Disorder; PHQ-9: Patient Health Questionnaire).

**Sample (*n* = 85)**	
**Demographics**	
Age	20.81 (±2.26)
Sex	72 female/13 male
GAD-7	5.12 (±3.47)
PHQ-9	5.69 (±2.93)
Compliance (%)	80.47 (±18.44)
**Affect**	
Forecasted PA-pomp	50.21 (±18.48)
Forecasted NA-pomp	18.71 (±11.76)
Experienced PA	55.60 (±18.46)
Experienced NA	22.06 (±12.26)

Forecasted and experienced PA (*r* = 0.45, *p* < 0.001) and NA levels (*r* = 0.43, *p* < 0.001) were significantly correlated, thus indicating a good degree of participants’ self-insight about future affect. Forecasted PA significantly predicted EMA-PA scores (*B* = 1.27, SD = 0.18, 95% CI [0.91, 1.63]; *p* < 0.001); similarly, forecasted NA significantly predicted EMA-NA scores (*B* = 0.94, SD = 0.18, 95% CI [0.58, 1.30]; *p* < 0.001).

Participants forecasted lower levels of PA than what they experienced (forecasted PA-POMP: mean = 50.21, SD = ±18.48; experienced PA: mean = 55.60, SD = ±18.46; *t* (84) = −2.57, *p* < 0.05). Similarly, a significant difference was observed between forecasted NA and experienced NA scores (forecasted NA-POMP: mean = 18.71, SD = ±11.76; experienced NA: mean = 22.06, SD = ±12.26; Z = −2.60, *p* < 0.01). Mean delta PA was -5.40 (SD = 19.37), whereas mean delta NA was -3.35 (SD = 13.29), thus indicating a general tendency to underestimate future affective states. No significant correlation was observed between delta PA and delta NA (*r* = −0.13, *p* = 0.25).

Participants with higher depressive symptoms anticipated to experience higher NA (*r* = 0.36, *p* < 0.001) and lower PA levels (*r* = −21, *p* = 0.05). However, delta values were not significantly associated with PHQ-9 scores (delta PA: *r* = −0.11, *p* = 0.30; delta NA: *r* = 0.20, *p* = 0.07), thus indicating that individuals with higher depressive symptoms forecasted and actually experienced lower levels of PA and higher levels of NA. Differently, forecasted NA (*r* = 0.65, *p* < 0.001) was significantly associated with anxiety symptoms, and delta NA significantly correlated with (*r* = 0.28, *p* < 0.01) and predicted delta NA [*R*^2^ = 0.11; *F*(1, 83) = 10.30; *B* = 0.83, SE = 0.03, 95% CI [0.03, 0.14]; *p* < 0.01], highlighting greater overestimation of future NA in the presence of increased anxiety symptoms.

### Affective Forecasting and Well-Being

Table 3 shows the association between psychological well-being measures and forecasted/experienced affect. Forecasted PA (self-acceptance: *r* = 0.53, *p* < 0.001; positive relations: *r* = 0.32, *p* < 0.01; autonomy: *r* = 0.43, *p* < 0.001; environmental mastery: *r* = 0.44, *p* < 0.001; personal growth: *r* = 0.42, *p* < 0.001; purpose in life: *r* = 0.42, *p* < 0.001) but not experienced PA significantly correlated with all Ryff’s subscales, revealing that participants holding more optimistic predictions of future PA reported greater psychological well-being. Additionally, forecasted NA showed a significant negative association with Ryff’s subscales of self-acceptance (*r* = −0.37, *p* < 0.001), autonomy (*r* = −0.27, *p* < 0.05), environmental mastery (*r* = −0.33, *p* < 0.01), and personal grow (*r* = −0.23, *p* < 0.01), while experienced NA did not correlate with any of the well-being measures.

**TABLE 3:** Correlations among all the variables included in the analyses.

	**1**	**2**	**3**	**4**	**5**	**6**	**7**	**8**	**9**	**10**	**11**	**12**	**13**	**14**	**15**	**16**
1. Forecasted NA	1.00	−	−	−	−	−⁣−	−	−	−	−	−	−	−	−	−	−
2. Forecasted PA	–0.12	1.00	−	−	−	−	−	−	−	−	−	−	−	−	−	−
3. Experienced NA	0.43***	–0.15	1.00	−	−	−	−	−	−	−	−	−	−	−	−	−
4. Experienced PA	0.04	0.45***	−0.22*	1.00	−	−	−	−	−	−	−	−	−	−	−	−
5. Delta NA	0.48***	–0.02	0.51***	0.20	1.00	−	−	−	−	−	−	−	−	−	−	−
6. Delta PA	–0.16	0.53***	–0.0	−52***	–0.13	1.00	−	−	−	−	−	−	−	−	−	−
7. PHQ-9	0.36***	−0.21*	0.13	–0.02	0.20	–0.12	1.00	−	−	−	−	−	−	−	−	−
8. GAD-7	0.65***	–0.16	0.30**	–0.06	0.28*	–0.19	0.68***	1.00	−	−	−	−	−	−	−	−
9. Self-acceptance	−0.37***	0.53***	–0.16	0.13	–0.16	0.33**	−0.43**	−0.45***	1.00	−	−	−	−	−	−	−
10. Positive relations	–0.19	0.32**	–0.01	0.00	–0.16	0.28**	–0.18	−0.23*	0.49***	1.00	−	−	−	−	−	−
11. Autonomy	−0.27*	0.43***	–0.16	0.16	–0.13	0.38***	−0.28**	−25*	0.53***	0.45***	1.00	−	−	−	−	−
12. Environment mastery	−0.33**	0.44***	–0.08	0.08	–0.18	0.26*	−0.37***	−0.41***	0.72***	0.57***	0.41***	1.00	−	−	−	−
13. Personal growth	−0.23*	0.42***	–0.05	0.06	–0.14	0.28*	–0.04	–0.07	0.54***	0.33***	0.45***	0.48***	1.00	−	−	−
14. Purpose in life	–0.16	0.42***	0.01	0.07	–0.12	0.33**	–0.19	−0.24*	0.66***	0.47***	0.38***	0.74***	0.61***	1.00	−	−
15. OFS	−0.21*	0.47***	–0.10	0.20	–0.06	0.25*	−0.30**	–0.20	0.59***	0.32***	0.40***	0.54***	0.47***	0.64***	1.00	−
16. CD-RISC	−0.27*	0.62***	−0.27*	0.19	0.05	0.37***	–0.14	−0.24*	0.53***	0.36***	0.46***	0.58***	0.55***	0.57***	0.60***	1.00

Forecasted PA, experienced PA, delta NA, delta PA, Ryff autonomy, Ryff environmental mastery and OFS were normally distributed, and correlations were calculated with Pearson correlations. The remaining associations were calculated using Spearman correlations. *p < 0.05, **p < 0.01, ***p < 0.001 (PHQ-9: Patient Health Questionnaire; GAD-7: Generalized Anxiety Disorder; OFS: Openness to Future Scale; CD-RISC: Connor-Davidson Resilience Scale).

Table 3 also shows the association between biased affective forecasting and psychological well-being. Delta PA was significantly correlated with all Ryff’s psychological well-being measures (self-acceptance: *r* = 0.33, *p* < 0.01; positive relations: *r* = 0.28, *p* < 0.01; autonomy: *r* = 0.38, *p* < 0.001; environmental mastery: *r* = 0.26, *p* < 0.05; personal growth: *r* = 0.28, *p* < 0.05; purpose in life: *r* = 0.33, *p* < 0.01): That is, positively biased PA forecasting was associated with enhanced perceived well-being. Consistently with our hypothesis, delta NA did not correlate with any of the well-being measures. When simultaneously included in a regression model to predict psychological well-being, delta PA was the only significant predictor of self-acceptance [*R*^2^ = 0.11; *F*(1, 82) = 5.21; delta PA: *B* = 0.10, SE = 0.03, 95% CI [0.03, 0.16]; *p* < 0.01; delta NA: *B* = −0.19, SE = 0.04, 95% CI [-0.11, 0.07]; *p* = 0.70], positive relations [*R*^2^ = 0.11; *F*(1, 82) = 5.11; delta PA: *B* = 0.09, SE = 0.03, 95% CI [0.02, 0.15]; *p* < 0.05; delta NA: *B* = −0.07, SE = 0.05, 95% CI [-0.17,.02]; *p* = 0.13], autonomy [*R*^2^ = 0.14; *F*(1, 82) = 6.91; delta PA: *B* = 0.10, SE = 0.03, 95% CI [0.04,.16]; *p* < 0.001; delta NA: *B* = −0.02, SE = 0.04, 95% CI [-0.10, 0.06]; *p* = 0.64], environmental mastery [*R*^2^ = 0.09; *F*(1, 82) = 3.97; delta PA: *B* = 0.05, SE = 0.02, 95% CI [0.01, 0.10]; *p* < 0.05; delta NA: *B* = −0.4, SE = 0.03, 95% CI [-0.11, 0.02]; *p* = 0.19], personal growth [*R*^2^ = 0.10; *F*(1, 82) = 4.53; delta PA: *B* = 0.08, SE = 0.03, 95% CI [0.03, 0.13]; *p* < 0.01; delta NA: *B* = −0.01, SE = 0.04, 95% CI [-0.09, 0.07]; *p* = 0.81], and purpose in life [*R*^2^ = 0.11; *F*(1, 82) = 4.94; delta PA: *B* = 0.09, SE = 0.03, 95% CI [0.03, 0.15]; *p* < 0.01; delta NA: *B* = −0.01, SE = 0.04, 95% CI [-0.09, 0.06]; *p* = 0.78].

Besides, forecasted PA (forecasted PA: *r* = 0.47, *p* < 0.001), and forecasted NA (*r* = −0.21, *p* = 0.05) were significantly associated with OFS. Additionally, only delta PA was significantly associated with OFS (*r* = 0.25, *p* < 0.05), suggesting that participants who overestimated future PA were more likely to report greater openness to the future.

Using GEEs, forecasted affect and EMA affect scores were simultaneously included as predictors of Ryff’s well-being measures (Table 4). Forecasted PA was the only significant predictor of positive relations (*B* = 0.54, SE = 0.08, 95% CI [0.09, 0.42]; *p* < 0.01), personal growth (*B* = 0.27, SE = 0.06, 95% CI [0.14, 0.39]; *p* < 0.001), and purpose in life (*B* = 0.31, SE = 0.07, 95% CI [0.17, 0.45]; *p* < 0.001), whereas both forecasted NA and forecasted PA significantly predicted self-acceptance (forecasted PA: *B* = 0.39, SE = 0.06, 95% CI [0.27, 0.51]; *p* < 0.001; forecasted NA: *B* = −0.33, SE = 0.09, 95% CI [-0.51, -0.15]; *p* < 0.001), autonomy (forecasted PA: *B* = 0.28, SE = 0.07, 95% CI [0.15, 0.41]; *p* < 0.001; forecasted NA: *B* = −0.24, SE = 0.11, 95%CI [-0.45, -0.02]; *p* < 0.05), and environmental mastery (forecasted PA: *B* = 0.24, SE = 0.05, 95% CI [0.14, 0.34]; *p* < 0.001; forecasted NA: *B* = −0.29, SE = 0.09, 95% CI [-0.47, -0.11]; *p* < 0.01). Interestingly, experienced daily affect did not predict any of the well-being measures.

**TABLE 4:** Generalized Estimating Equation (GEE) models introducing forecasted PA, forecasted NA, daily EMA-PA and daily EMA-NA as predictors of well-being subscales.

	**Self-acceptance**		**Positive relations**		**Autonomy**		**Environmental mastery**		**Personal growth**		**Purpose in life**	
	***B***	***SE***	***B***	***SE***	***B***	***SE***	***B***	***SE***	***B***	***SE***	***B***	***SE***
**Coefficients**												
Forecasted PA	0.39***	0.06	0.25**	0.08	0.28***	0.07	0.24***	0.05	0.27***	0.06	0.31***	0.07
Forecasted NA	-0.33***	0.09	-0.24	0.14	-0.24*	0.11	-0.29**	0.09	-0.16	0.09	-0.15	0.11
Daily PA	0.000	0.002	-0.001	0.001	-0.001	0.001	0.001	0.0003	0.000	0.001	-0.001	0.001
Daily NA	-0.002	0.002	0.000	0.001	0.000	0.001	<0.001	0.0002	-0.001	0.001	0.000	0.001

B = unstandardized regression coefficient; *p < 0.05, **p < 0.01, ***p < 0.001 (PA: Positive affect; NA: Negative affect).

### Affective Forecasting, Resilience and Stress

Forecasted PA (*r* = 0.62, *p* < 0.001) and delta PA (*r* = 0.37, *p* < 0.001) but not experienced PA (*r* = 0.19, *p* = 0.08) significantly correlated with CD-RISC. That is, holding optimistic expectations regarding the future and overestimating PA were associated with higher levels of resilience. Besides, forecasted (*r* = −0.27, *p* = < 0.05) and experienced NA (*r* = −0.27, *p* < 0.05) but not delta NA (*r* = 0.05, *p* = 0.67) did show a significant association with resilience (Table 3).

Resilience was a significant positive predictor of psychological well-being (self-acceptance: *R*^2^ = 0.32; *F*(1, 83) = 39.87; *B* = 0.48, SE = 0.08, 95% CI [0.33, 0.63]; *p* < 0.001; positive relations: *R*^2^ = 0.14; *F*(1, 83) = 13.54; *B* = 0.34, SE = 0.09, 95% CI [0.16, 0.52]; *p* < 0.001; autonomy: *R*^2^ = 0.21; *F*(1, 83) = 21.82; *B* = 0.35, SE = 0.07, 95% CI [0.20, 0.50]; *p* < 0.001; environmental mastery: *R*^2^ = 0.36; *F*(1, 83) = 46.93; *B* = 0.38, SE = 0.06, 95% CI [0.27, 0.49]; *p* < 0.001; personal growth: *R*^2^ = 0.28; *F*(1, 83) = 32.22; *B* = 0.38, SE = 0.07, 95% CI [0.28, 0.52]; *p* < 0.001; purpose in life: *R*^2^ = 0.35; *F*(1, 83) = 44.22; *B* = 0.46, SE = 0.07, 95% CI [0.32, 0.59]; *p* < 0.001). The inclusion of delta PA significantly increased the variance explained by the model for autonomy (*R*^2^ = 0.62, Δ*R*^2^ = 0.05, *F*(2, 82) = 14.57, CD-RISC: *B* = 0.28, SD = 0.08, 95% CI [0.13, 0.44]; *p* < 0.001; delta PA: *B* = 0.07, SD = 0.03, 95% CI [0.01, 0.12]; *p* < 0.05), and a close-to-significance trend was observed in the model predicting positive relations (*R*^2^ = 0.17, Δ*R*^2^ = 0.03, *F*(2, 82) = 8.40, CD-RISC: *B* = 0.28, SD = 0.10, 95% CI [0.09, 0.47]; *p* < 0.001; delta PA: *B* = 0.06, SD = 0.03, 95% CI [-0.01, 0.13]; *p* = 0.08).

Finally, GEE analyses were conducted to explore whether EMA-stress scores and delta PA significantly predicted EMA-affect. EMA-NA was significantly predicted by EMA-stress level but not by delta PA (EMA-stress: *B* = 0.46, SD = 0.02, 95% CI [0.42, 0.51]; *p* < 0.001; Delta PA: *B* = −0.04, SD = 0.04, 95% CI [-0.11, 0.03]; *p* = 0.26), thus underlying the fundamental role of stress on NA affect ratings (i.e., the experience of higher stress was associated with higher levels of perceived NA). Similarly, EMA-stress scores but not delta PA significantly predicted positive emotion level (stress: *B* = −0.09, SD = 0.01, 95% CI [-0.11, -0.08], *p* < 0.001; Delta PA: *B* = −0.02, SD = 0.02, 95% CI [-0.07, 0.03], *p* = 0.46). Notably, a significantly different association between EMA-NA and stress was observed as a function of delta values (stress: *B* = 0.45, SD = 0.02, 95% CI [0.40, 0.50], *p* < 0.001; Delta PA: *B* = 0.02, SD = 0.04, 95% CI [-0.05, 0.09], *p* = 0.60; interaction: *B* = −0.003, SD = 0.001, 95% CI [-0.01, 0.00]; *p* < 0.05). As indicated by the negative beta coefficient of the interaction (Suso-Ribera et al., 2019), as delta PA becomes more positive (i.e., future PA is overestimated), the contribution of stress on NA is reduced. A significantly different association between EMA-positive emotion and stress was also observed as a function of PA delta values (stress: *B* = −0.09, SD = 0.01, 95% CI [-0.10, -0.07], *p* < 0.001; Delta PA: *B* = −0.04, SD = 0.03, 95% CI [-0.09, 0.01], *p* = 0.12; interaction: *B* = 0.001, SD = 0.0003, 95% CI [0.001, 0.002]; *p* < 0.001). As the interactive effect of delta PA and stress on positive emotion level is positive, this means that, as delta PA becomes more negative (i.e., forecasting becomes more negatively biased and future PA is underestimated), stress becomes more deleterious for positive emotions. In other words, it is possible to suggest that, despite the increase in experienced stress, subjects with positively biased PA forecasting (i.e., those who overestimated future positive affective states) reported lower NA levels and higher positive emotions.

Regarding delta NA, EMA-PA (stress: *B* = −0.36, SD = 0.03, 95% CI [-0.42, -0.30], *p* < 0.001; Delta NA: *B* = 0.14, SD = 0.11, 95% CI [-0.07, 0.36], *p* = 0.19) was significantly predicted by EMA-stress but not delta NA, whereas EMA-positive emotions were significantly predicted by both stress level and delta NA (stress: *B* = −0.14, SD = 0.02, 95% CI [-0.18, -0.09], *p* < 0.001; Delta NA: *B* = 0.51, SD = 0.16, 95% CI [0.83, 0.19], *p* < 0.01). No significant interaction effect was observed.

## Discussion

So far, a growing body of literature has explored people’s ability to forecast emotional experiences in relation to specific future events. In the current study, instead, we investigated affective forecasting as a future-oriented disposition, asking participants to estimate their affect during a 2-week period.

The main aim of the present study was to explore whether biased affective forecasting was associated with perceived psychological well-being, consistently with the hypothesis that the ability to estimate future emotional experiences constitutes a future-oriented strategy to regulate emotions (Goodhart, 1985; Totterdell et al., 1997).

Aligned with the previous literature (Buehler and McFarland, 2001; Wirtz et al., 2003), participants in the present study showed a good degree of insight about their future PA and NA levels. A significant discrepancy between forecasted and experienced affect was also observed, and participants showed a somewhat pessimistic view of the future. These results diverge from what has been revealed by a growing body of literature exploring affective forecasting in relation to specific future events. People would indeed overestimate the impact of both positive and negative future events (Wilson and Gilbert, 2003), due to an excessive focus on a single event in isolation without considering the general context and background distractions (Wilson et al., 2000). This phenomenon, called *focalism*, does not occur when forecasting general emotional states, which could explain the dissimilar results observed in this study. Besides, our results confirmed the role played by depressive and anxiety symptoms on affective forecasting (Wenze et al., 2012), and the presence of mild symptoms was associated with a negative bias, which is consistent with the previous literature (Craske and Pontillo, 2001; Gotlib and Joormann, 2010; Colombo et al., 2019c). Specifically, depressive symptoms were associated with future NA overestimation and PA underestimation, whereas anxiety symptoms only significantly correlated with future NA overestimation. As evidenced by the tripartite model, indeed, depression and anxiety share the same pattern of enhanced NA, whereas low levels of PA and anhedonia are only typical of depressive conditions (Clark and Watson, 1991).

Coherently with the first hypothesis, participants holding more positive estimations of future PA and positively biased PA forecasting reported greater psychological well-being on almost all Ryff’s subscales. Results also confirmed the hypothesis that biased NA estimations (i.e., underestimating or overestimating NA) were not significantly associated with well-being, which supports the idea that a bias in negative affective forecasting does not affect psychological well-being. Besides, it is of particular interest that psychological well-being was significantly predicted by forecasted but not experienced affect. In other words, our results suggest that psychological well-being is a grounded dimension: Rather than momentary affect and daily events, psychological well-being seems to be more strongly associated with resilience and coping skills, such as holding an optimistic, even if distorted, vision of the future. Accordingly, delta PA but not delta NA was significantly associated with OFS, which in turn has been found to be associated with better mental health (Botella et al., 2018).

Our results also confirmed the second hypothesis. Contrary to delta NA, forecasted PA as well as delta PA were strongly associated with resilience, and participants holding more positive estimations of future PA and overestimating future PA were found to be more resilient. The multiple regression analyses also showed that delta PA in addition to resilience improved the prediction of some well-being dimensions, thus confirming the idea that positively biased affective forecasting may constitute a coping skill that increases individuals’ abilities to deal with daily hassles. Consistently, and confirming our third hypothesis, delta PA significantly moderated the impact of daily stress on daily affect. This means that, when experiencing high levels of stress, subjects who tended to overestimate future PA reported lower NA and higher positive emotions than subjects who showed a tendency to underestimate it. A positive attitude toward the future seems therefore to be an adaptive coping resource in highly stressful situations, allowing to maintain better levels of momentary affect despite the presence of intense stressors.

Even though a long tradition of research considered cognitive distortions as maladaptive mechanisms associated with worse mental-health (Jahoda, 1953), there is now increasing evidence revealing that, in certain circumstances, cognitive biases may rather be adaptive (Taylor and Brown, 1988). Specifically, people’s perception of the future has been shown to affect mental health (Weinstein, 1980; Mikus et al., 2017), and openness to the future has been associated with higher positive emotions, psychological well-being, and self-esteem (Botella et al., 2018). This seems to be strictly connected to the construct of optimism, defined as “[…] *a mood or attitude associated with an expectation about the social or material future*” (Tiger, 1979), which has been shown to increase people’s skills to deal with challenging events (Carver et al., 2012) and to be associated with higher subjective well-being, health and life success (Forgeard and Seligman, 2012). Beyond the conceptualization of optimism as an explanatory style (Seligman, 1991), the definition of optimism as one’s disposition to hold favorable or unfavorable expectations and beliefs about the future seems to be more coherent with our results (Carver and Scheier, 2014). In this regard, we suggest that positively biased affective forecasting may in part reflect one’s dispositional optimism, and it may constitute a mechanism that increases people’s skills to deal with daily events, thus having a positive impact on psychological well-being.

Although optimism toward the future is likely to foster coping skills and promote well-being, it is important to note that holding a positively biased view of reality can also be maladaptive in certain circumstances (Chang et al., 2009). For instance, there is evidence showing that optimistic individuals are more likely to show gambling behaviors (Gibson and Sanbonmatsu, 2004) or to report lower motivation when trying to quit smoking (Weinstein et al., 2004). As suggested by Forgeard and Seligman (2012), “*the most adaptive outlook seems therefore to be mostly optimistic, tempered with small doses of realistic pessimism when needed*”: For example, to avoid disappointment when idealizing something that it is quite improbable to achieve. A flexible rather than rigid positively biased perspective seems therefore to be the key of well-being. Future research should investigate the potential role of flexibility on affective forecasting and health-related outcomes.

Besides, the findings of the current study have to be considered in light of some limitations. In the present study, we excluded individuals with clinically relevant depressive and anxiety symptoms in order to control for the confounding effect of a pathological negative bias (Wenze et al., 2012; Mathersul and Ruscio, 2019). However, there are other individual factors, which have been shown to play a fundamental role in affective forecasting abilities. For example, personality has been found to explain 30% of the concordance between anticipated and experienced emotional experiences (Zelenski et al., 2013; Hoerger et al., 2016), and introverted as compared to extroverted individuals tend to anticipate more unpleasant emotions and less positive emotional states. Furthermore, there is evidence showing that people who are high in emotional intelligence are more accurate at encoding and predicting their emotional reactions (Dunn et al., 2007b; Hoerger et al., 2012). Altogether, these results suggest that affective forecasting is a complex cognitive phenomenon, in which many different factors are likely to reciprocally interact with each other. In addition to the previous, it is also possible to hypothesize that an individual’s response style to positive emotional states (Feldman et al., 2008) could be an additional element that influences positive affective forecasting. Accordingly, habitual positive ruminators (i.e., those who tend to reflect on positive events, self-qualities, and pleasant emotions) might be more likely to be positively biased toward their future emotional states, as a result of an over-focus on positive emotional experiences and/or qualities. Future research is needed to prove this hypothesis and, more generally, to build a broader framework in which all the aforementioned factors are concurrently considered.

It is also important to note that the sample was mainly composed of undergraduate female students. Future research should investigate whether other factors such as sex or age may entail different effects on affective forecasting. To date, elderly people as compared to young individuals have been shown to recall more positive than negative information, a phenomenon called positivity effect (Reed and Carstensen, 2012; Carstensen and DeLiema, 2018). However, this positivity effect does not seem to influence elderly’s affective forecasting (Nielsen et al., 2008), who have been shown to be accurate rather than positively biased in the estimation of future affective states, thus suggesting that “[…] *people may correct for this bias as they age*.” Accordingly, the results observed in our study may not be generalizable to all populations, and it is possible that positively biased estimations of future states are more common in young-to-middle adulthood.

Additionally, the methodological nature of this study only allows to draw correlational conclusions, and more evidence is needed to clarify the potential causal role of biased forecasting on perceived well-being and resilience. Hence, experimental designs could complement existing evidence assuming causal inferences. Future studies should also consider the potential consequences of this cognitive bias on behaviors, exploring whether holding positive expectations about future emotions may also affect people’s decisions in daily life. It might be possible, indeed, that biased affect predictions influence daily behavioral attitudes (such as avoiding or joining specific situations), which in turn may influence well-being. Finally, the use of single items to measure EMA-PA and EMA-NA might not capture the complexity of momentary affect, as opposed to the use of the PANAS for the assessment of affective forecasting. However, we decided to use single items in order to reduce participants’ burden and increase adherence rates (Colombo et al., 2018, 2019c), similarly to previous studies (Suso-Ribera et al., 2018). Besides, the autonomy and environmental mastery Ryff’s subscales showed low internal consistency, and analyses including both measures have to be taken with caution.

To conclude, the benefits of enhancing PA as a way to promote mental health and well-being has been widely supported (Pressman et al., 2018), thus suggesting the importance of developing specific interventions to potentiate people’s strategies to regulate positive emotions. In particular, it is of utmost importance to clearly determine the importance of developing a positive bias as well as an optimistic rather than pessimistic attitude toward the future. Besides, it is arguable that the complex dynamic of emotional and cognitive processes that intrinsically conform the regulatory process of individuals does not need evaluative precision but rather intrinsic coherence that the future will be possible to cope with.

## Data Availability Statement

The data that support the findings of this study are openly available in OSF at https://doi.org/10.17605/OSF.IO/JFS3K (Colombo et al., 2020).

## Ethics Statement

This study was approved by the ethics committee of the Jaume I University (Spain) (certificate number: CD/57/2019; reference: 41EA95C7D3C8747F0A37), and informed consent was obtained from all participants. The patients/participants provided their written informed consent to participate in this study.

## Author Contributions

DC contributed to the conception and design of the work; the acquisition, analysis and interpretation of data; and the drafting the manuscript. JF-Á and CS-R equally contributed to the critical revision of the work. PC, AG-P, CB, and GR contributed to writing—review processes, editing and supervision.

## Conflict of Interest

The authors declare that the research was conducted in the absence of any commercial or financial relationships that could be construed as a potential conflict of interest.
